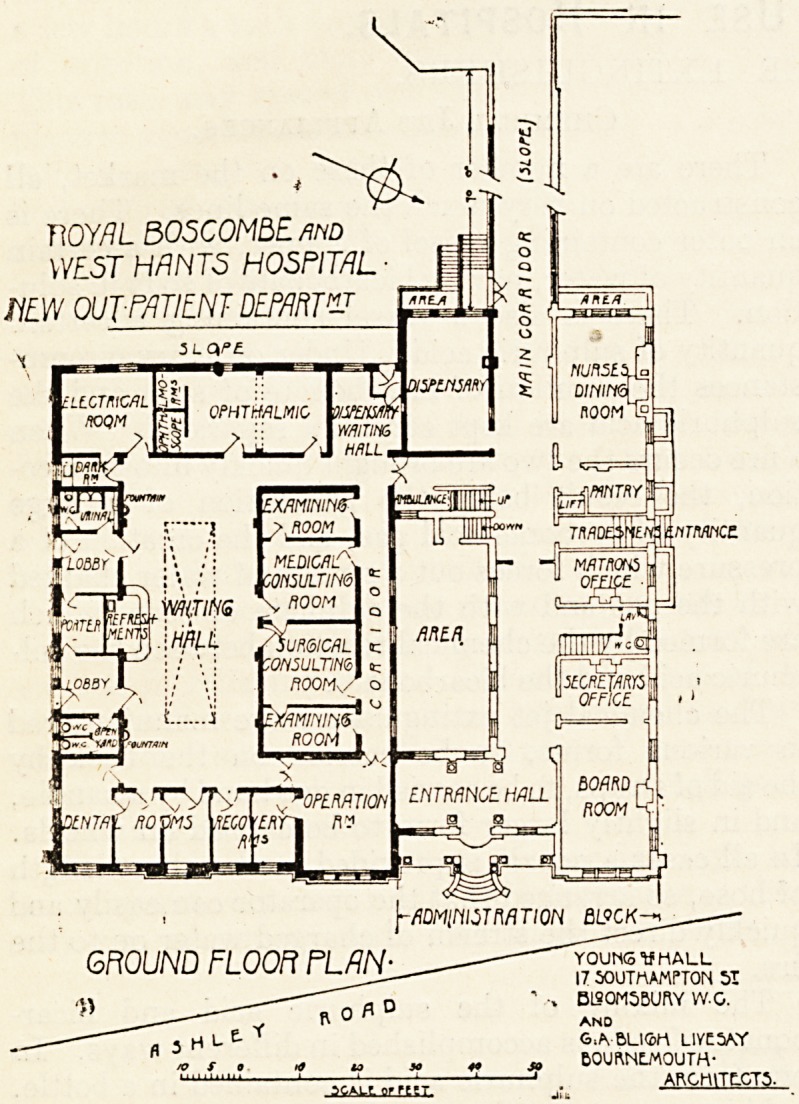# Royal Boscombe and West Hants Hospital

**Published:** 1909-05-29

**Authors:** 


### ROYAL BOSCOMBE AND WEST HANTS HOSPITAL.

For some years the work of this hospital has been carried on in buildings partly old and partly new, the new buildings being wards and their appurtenant offices and admirably fitted for their work, while the old building provided inadequately for the staff, the administrative offices, and the out-patient work.

The ground-floor plan which we publish to-day shows the beginning that has been made towards the gradual removal of this inconvenient state of things by providing an ample and suitably equipped building for the work of the out-patient department. This building, while complete in itself so far as the department is concerned, will ultimately form part of a larger building, in which the resident medical staff, the matron, and the kitchen offices will be housed; while it is hoped at some future time to erect at the south side of the entrance for out-patients a suitable Nurses' Home.

The entrance for out-patients is on the south side, and is approached by a carriage-road from Ashley Road. Eventually a covered shelter will be erected protecting both entrances, and also connecting the Nurses' Home with the out-patient department, and so with the wards. There are two separate entrances—one for male, the other for female patients—and between them is placed the porter's entrance, which communicates by both doors and small windows with each entrance. Inside the porter's office is the main switchboard for the electric light, every point of which is thus under his control. Communicating with each lobby are the necessary sanitary offices, which are cut off from the rest of the building by open yards. From the lobbies patients pass into the large waiting-hall, around which are arranged the various consulting-rooms, etc.

At the south end of the waiting-hall is a refreshment bar. The medical and surgical rooms, each with their examining-rooms attached, are on the north side, and a passage at the back of these leads to the operation-room on the one hand and to the medicine waiting-room on the other, so that patients from these rooms do not pass back into the waiting-hall. On the east side are three rooms for dental surgery and mechanical work, two recovery-rooms and the operation-room. On the west side is the electrical room and the ophthalmic room. This room is also used for the ear and throat department. It is provided with two dark-rooms. A small waiting-room for medicine adjoins the dispensary, and out of this is the exit lobby for patients. The dispensary is arranged to serve both in-patients and out-patients.

The walls generally inside have a dado of white glazed brick with a green line above, and are finished above with Keene's cement and Hall's sanitary paint. The floors are of marble terrazzo throughout. The new buildings have been designed and carried out under the supervision of Messrs. Young and Hall, of London, and Mr. G. A. Bligh Livesay, of Bournemouth.

## Figures and Tables

**Figure f1:**